# The Legacy of Sickness Behaviors

**DOI:** 10.3389/fpsyt.2020.607269

**Published:** 2020-12-03

**Authors:** Keith W. Kelley, Stephen Kent

**Affiliations:** ^1^Department of Pathology, College of Medicine, Urbana, IL, United States; ^2^Department of Animal Sciences, College of Agricultural, Consumer & Environmental Sciences (ACES), University of Illinois at Urbana-Champaign, Urbana, IL, United States; ^3^School of Psychology and Public Health, University of Illinois in Urbana-Champaign, Urbana, IL, United States; ^4^School of Psychology and Public Health, La Trobe University, Melbourne, VIC, Australia; ^5^Dean and Head of School of Psychology & Public Health, Melbourne, VIC, Australia

**Keywords:** immune-brain communication, fever, systemic physiology, interleukin-1, motivated behaviors

## Abstract

Systemic infections of all types lead to a syndrome known as sickness behaviors. Changes in the behavior of febrile humans and animals formed the original basis for this concept. Body temperature is behaviorally regulated in both endotherms and ectotherms. However, infections cause other changes in body functions, including sleep disruption, anorexia, cognitive and memory deficits and disorientation. The brain mediates this entire cluster of symptoms, even though most major infections occur outside the brain. The true importance of sickness behaviors is not the numerous discoveries of symptoms that affect all of us when we get sick. Instead, the legacy of 30 years of research in sickness behaviors is that it established the physiologic importance of reciprocal communication systems between the immune system and the brain. This conceptual advance remains in its infancy.

## Introduction

It started with a fever. Well, really, it started with trying to understand fever. After all, pathogenic microbes like SARS-CoV-2 infect epithelial cells in the lungs as well as endothelial cells. Influenza also infects epithelial cells of the respiratory tract. Entry of SARS-CoV-2 and influenza into these cells establishes residence, as well as their ability to set up housekeeping outside the brain. Yet, a part of the brain known as the hypothalamus and higher brain centers control fever, not the lungs. This finding characterizes the research on fever and infectious diseases that long followed disparate paths. Lipopolysaccharide (LPS) is composed of a lipid and a polysaccharide expressed on the outer membrane of Gram-negative bacteria. Following the discovery that LPS acts as an adjuvant to enhance antibody responses, graduate students who studied immunology in the 1960s, 70s, and ‘80s were taught that the main target for LPS is B lymphocytes. This of course ignored the much earlier discovery in 1888 that injection of heat-killed gram-negative bacteria into rabbits causes fever (summarized by Cavaillon; https://www.lpsbiosciences.com/index.php/news-blog-3/lps-history). This simple example exemplifies the dichotomy in the relationship between studies in immunology and systemic physiology that continued until the end of the twentieth century.

This unrealistic state of affairs began to collapse in the late 1970s as a consequence of the purification, cloning and expression of the first cytokine, human interferon-α, in 1978. Six years later, the United States Food and Drug Administration approved interferon-α for clinical use. Advances in recombinant DNA technology and expression systems rapidly led to purification and sequencing of two more “substances” produced by leukocytes. Their biological activities defined their names: endogenous pyrogen and/or lymphocyte activating factor and T-cell growth factor. Time-consuming and costly biological assays were required for quantification. ELISA assays did not exist. The scientific community now defines these two substances as interleukin-1 (IL-1) and interleukin-2 (IL-2), respectively. Thirty-five more cytokines and their receptors have been sequenced, cloned and expressed since the original discovery of IL-1 and IL-2.

All modern-day physiologists, immunologists, and neuroscientists who are engaged in temperature regulation research are now fully aware that in order to understand fever one needs to understand cytokines. They know that pathogens like gram-negative bacteria, SARS-Cov-2 and influenza lead to production of endogenous pyrogens in the periphery and the hypothalamus. In turn, these endogenous pyrogens alter the activity of warm- and cold-sensitive neurons in the preoptic area of the anterior hypothalamus. The result is the production of second messengers like prostaglandin E2 (PGE 2) and ceramide that initiate physiological changes leading to fever. But these scientists still want to know how something produced outside the brain communicates with structures inside the brain to cause fever. Circumventricular organs that possess permeable capillaries, blood-borne cytokines that activate cerebral microvascular endothelial cells, afferent neural connections from the periphery to the brain like the vagus nerve, entry of immune cells into the brain parenchyma via the choroid plexus and the recently discovered lymphatic drainage system in the meninges all have scientific support. But a precise and universally accepted roadmap of all of the specific routes of the communication networks that mediate not only fever but also the variety of other symptoms that accompany sickness still does not exist.

## Groundwork for the Discovery of Sickness Behaviors

### Something From the Immune System Increases Glucocorticoids in Blood

Hans Selye advanced the concept of stress by showing that a variety of discomforting challenges in rats cause hypertrophy of the adrenals and involution of the thymus gland, one of the two primary lymphoid organs where lymphocytes are formed (1). Knowledge of the immune system was in its infancy at that time, but this discovery pointed scientists to a potential relationship between stress and immunity that occurred via a route that was later shown to travel through the brain (i.e., the hypothalamic-pituitary-adrenal axis). By the 1950s, the scientific community accepted that a part of the brain known as the hypothalamus was critical to stress responses. Noxious stimuli cause the hypothalamus to secrete corticotropin-releasing factor (CRF) into hypophyseal portal vessels that connect to the anterior pituitary gland. This causes release of pituitary-derived adrenocorticotropin hormone (ACTH) into the blood. Within an hour, there is an increase in systemic concentrations of the glucocorticoids corticosterone (rodents) and cortisol (humans). These early discoveries defined the hypothalamic-pituitary-adrenal axis.

In 1957, Wexler et al. established that injection of LPS peripherally causes an increase in plasma glucocorticoids (2). This led to the heretical idea that foreign antigens not only induce an immune response but also inform the brain that the body is experiencing an infection. But exactly how this occurred had to await the discovery of immune messengers that were cloned and expressed in the late 1970s and early 1980s. We now know these proteins as cytokines.

### Systemic IL-1 Increases Both Adrenocorticotropic Hormone and Glucocorticoids

Besedovsky et al. reported the earliest and strongest evidence to establish that cytokines act as afferent signals that carry messages to the brain. His group injected supernatants from mitogen-stimulated spleen cells into the peritoneum of rats. This caused corticosterone in blood to rise (3) and norepinephrine in the hypothalamus and brain stem to decline (4). Professor Besedovsky, however, was not only a physiologist but also a scholar of immunology. He knew that glucocorticoids not only inhibit many actions of the immune system but were likely to also impair the production of the newly-discovered cytokine IL-1. His expertise in physiology led him to speculate that a negative feedback system existed between the immune system and the anterior pituitary gland (5). In this model, IL-1 released after a systemic infection would cause secretion of ACTH, a rise in plasma glucocorticoids and inhibition of the initiating immune response. He tested this hypothesis by isolating human white blood cells and exposing them to Newcastle disease virus. Supernatants from these cultures increased plasma glucocorticoids. Interferon-α was likely secreted into these supernatants. However, interferon-α is species-specific, so human interferon-α was unlikely to be the culprit when injected into rodents. Instead, he attempted to block the glucocorticoid-increasing activity by pretreating the supernatants with a neutralizing antibody to IL-1. This experiment established that supernatants treated with the IL-1 neutralizing antibody were unable to increase plasma glucocorticoids in rats. Professor Besedovsky went on to show that recombinant IL-1 increased corticosterone in nude mice that lack T cells. As such, the glucocorticoid-stimulating activity of IL-1 was unlikely to be a downstream product of T cell-derived cytokines such as IL-2. These experiments proved that IL-1 serves as an afferent signal from the immune system that subsequently increases both pituitary-derived ACTH and corticosterone from the adrenal gland.

### Systemic IL-1 Acts in the Brain

The experiments described above were clever and ground-breaking, but no structure in the brain had yet been shown to be involved. Neurons from a part of the brain known as the paraventricular nucleus of the hypothalamus secrete CRF that causes release of pituitary-derived ACTH into the blood. But other possible sources of ACTH exist, such as a report showing that IL-1 induces release of ACTH directly from pituitary cells (6) and expression of the ACTH precursor gene, proopiomelanocortin, by leukocytes (7). Two back-to-back publications in *Science* magazine unequivocally proved that systemic administration of IL-1 increased release of CRF from hypothalamic neurons (8, 9). These data convincingly proved that soluble substances produced by activated leukocytes could inform the brain that a perturbation has occurred in the immune system. This groundbreaking finding led others to ask if these same signals from the immune system affect other aspects of brain function, with a particular emphasis on behavior.

## Sickness Behaviors

### Thermoregulatory Behavior Formed the Basis of the Concept of Sickness Behavior

The concept of sickness behaviors had its roots in a large literature on thermoregulatory behavior. By the 1980s, it was well-known that both endo- and ectotherms utilize a variety of behaviors to regulate body temperature. For example, ectotherms like reptiles estivate during the heat of the day to reduce water loss and prevent pathological damage caused by high body temperature. Endotherms like mammals and birds lower their metabolic load in hot weather by reducing foraging for food and seeking cooler environments (e.g., burrows or shade). In cold weather, both ectotherms and endotherms reduce their surface area to minimize heat loss, a behavior that often takes the form of huddling.

Benjamin Hart, a veterinarian at the University of California, Davis wrote a review article in which he connected the dots between fever and behavior (10). He noted that striking changes in behavior occur during fever, including reduced food consumption, animal grooming to promote evaporative heat loss and polydipsia, lethargy and insomnia. Professor Hart wrote, “…the behavior of sick animals and people is not a maladaptive response or the effect of debilitation, but rather an organized, evolved behavioral strategy to facilitate the role of fever in combating viral and bacterial infections.” His paper extended ideas about fever to a variety of new and some previously unrecognized thermoregulatory behaviors. This review reshaped the landscape about immune system to brain communication. However, the focus was on fever and thermoregulatory behavior. It did not address the multiple aspects of sickness behaviors recognized today. These include symptoms such as inflammatory pain, a variety of mental health disorders and learning and memory deficits.

All new theories and concepts must be rigorously tested before they become facts. Cloning and expression of cytokines like IL-1, tumor necrosis factor (TNF), and IL-6 occurred in the early part of the 1980 decade. But most of the behavioral experiments with these recombinant cytokines were initially aimed at investigating their role as endogenous pyrogens that elevate the hypothalamic set point and lead to fever. Data from these experiments ultimately led to the realization that both IL-1 and TNF serve as endogenous pyrogens. However, motivated behaviors classically reside in other structures of the limbic system beside the hypothalamus. As such, the question arose as to whether cytokines would affect other brain regions like the limbic cortex, hippocampal formation and amygdala. In short, the Hart review that focused on the connection between disease and fever encouraged scientists to ask whether other behaviors induced by infections and the subsequent release of cytokines are mediated by higher-order brain structures.

#### Reduction in Motivated Behavior Is Independent of Fever

The first clue that cytokines are involved in human sickness behaviors came from phase 1 clinical trials for cancer. Systemic injections of interferon α, IL-1 and IL-2 all induced a variety of adverse effects, including inappetence, fever, headaches, fever, malaise, disorientation and somnolence (11). Many scientists argued that these CNS-mediated symptoms were due to toxicity caused by injection of high doses of cytokines into very sick patients. Clearly, these early data established that peripheral cytokines could have major effects on the brain. However, scientists considered the effects of systemic cytokines on the brain to be pure pharmacological rather than physiological effects. They did not seriously consider the possibility that physiologic concentrations of peripheral cytokines synthesized and released following exposure to infectious and non-infectious agents could communicate with the brain to induce sickness behaviors.

Professor Evelyn Satinoff at the University of Illinois Urbana-Champaign was a pioneer in the field of thermoregulation, which led her to investigate fever, infection and sleep and wakefulness. Stephen Kent earned his Doctorate of Philosophy under her guidance. In 1990, Steve accepted a post-doctoral position to work with both Professors Kelley and Dantzer using behavioral equipment that was available in Bordeaux, France. Steve designed and conducted clever experiments and published the data with Nancy Rothwell's group in Manchester, England. The goal was to determine if fever is responsible for the IL-1-induced reduction in food-motivated behavior (12). He used classic operant conditioning chambers in which hungry rats had to press a lever to obtain a small pellet of food. Rats were given an injection of recombinant IL-1 systemically (intraperitoneal, IP) or centrally (intracerebroventricular; ICV). Injection of the IL-1 receptor antagonist (IL-1RA) preceded injections. Food-motivated behavior, social investigation of a novel juvenile, body temperature and metabolic rate were the dependent variables.

As expected, IL-1 administered in either the peritoneum or brain ventricles increased body temperature (~1.5 C) and oxygen consumption (~18%) and reduced food-motivated behavior (~90%) and social investigation (~90%). Pretreatment with IL-1RA systemically followed by IL-1 given via the same peritoneal route reduced both body temperature and metabolic rate. However, when both compounds were administered ICV, IL-1RA failed to affect either of these two variables. The behavioral experiments provided considerably different results. Regardless of whether IL-1 was given IP or ICV, the IL-1RA antagonist injected by the same route blocked both the reduction in food-motivated behavior and social investigation. Pretreatment with IL-1RA given ICV followed by injection of IL-1 via the IP route yielded an unexpected result. Although the antagonist had no effect on either the rise in body temperature or metabolic rate, it blocked the reduction in both food-motivated and social behavior. This result established that rats with an elevated metabolic rate and fever are fully capable of engaging in motivational behaviors. As such, the data were interpreted to indicate that the fever-inducing and the behavioral effects of IL-1 are medicated by different receptor mechanisms, all of which was summarized in a state-of-the-art review article (13). Subsequently, Fortier et al. (14) demonstrated that the febrile and anorexic effects of the viral mimic, polyinosinic:polycytidylic acid (poly I:C), are mediated via differing pathways. Similarly, Damm et al. (15) reported that LPS-induced sickness behaviors and fever can be disassociated. Importantly, Corrard et al. (16) reported similar results in feverish children with data showing a dissociation between a number of clinical sickness behaviors and the severity of fever. These human data are consistent with the original results of Kent et al. (12) by showing that clinical manifestations of sickness are independent of fever.

Experiments published more than 20 years after the initial reports of sickness behaviors have strengthened these early findings showing that fever can occur separately from sickness behaviors. For example, investigators interested in motivational theory have studied sickness behaviors. They asked whether physiological states like hunger, fear or libido affect any aspect of sickness behaviors. They found that sickness can either increase or decrease social behaviors [reviewed by (17)]. A recent paper reported that sickness behaviors and fever can be disassociated in LPS-injected guinea pig pups, depending upon whether LPS is injected with their mother nearby (18). Presence of the mother enhanced LPS-induced fever in the pups even though sickness behaviors were nearly absent. In humans, activation of the immune system with a typhoid vaccine increased negative mood and IL-6 in the absence of fever (19). Once again, exogenous stimuli as provided by stressful psychological tasks increased these differences, consistent with theories of motivation.

## CODA

Prior to the beginning of the twenty-first century, naysayers argued that the immune and central nervous systems do not dialogue with one another. They advocated maintaining the distinct disciplines of immunology and neuroscience, with little to no communication between the two. But emerging data demanded a more innovative approach. Physiology, which is a truly integrative science that spans reciprocal regulatory control systems among all organ systems, was not seriously considered. The major reasons for the arguments of skeptics were the existence of the blood-brain-barrier (BBB), lack of CNS lymphoid vessels and paucity of antigen-presenting cells in the brain. The scientific community now accepts that the BBB is much more than a barrier, acting as a true interface between the blood and brain [BBI; (20)]. Secondly, a century of science was turned upside down by the discoveries of Louveau et al. (21) and Aspelund et al. (22) who showed convincing histological evidence and now the functional importance of the meningeal lymphatic system [reviewed by (23)]. Finally, it is well-documented that microglia, monocytes and dendritic cells in the brain parenchyma can express major histocompatibility antigens that present antigen to T lymphocytes [reviewed in (24)]. And of course the discovery and identification of 37 cytokines, their receptors and multiple chemokines ushered in a entirely new way of thinking about immune-brain networks.

As shown by the early experiments on sickness behaviors, systemic cytokines alert the brain that insults such as an infection or trauma have occurred in the periphery. Indeed, the brain can synthesize and express several cytokines. Many other fascinating discoveries have provided entirely new insights into brain-communication systems. They range from links between clinical depression and systemic inflammation [see reviews by (25, 26)] to the emerging roles of neurotransmitters such as acetylcholine and catecholamines in the development of bioelectronic medicine for treatment of diseases like rheumatoid arthritis and Crohn's disease [reviewed in (27)]. As such, nearly all of the naysayers have disappeared. The discovery of sickness behaviors in all forms established the powerful role of communication between the immune system and the brain. This is the true legacy of sickness behaviors.

## Author Contributions

The concept for this article was developed and proposed by KK and the article was jointly prepared by KK and SK. All authors contributed to the article and approved the submitted version.

## Conflict of Interest

The authors declare that the research was conducted in the absence of any commercial or financial relationships that could be construed as a potential conflict of interest.
